# The learning curve for robotic-assisted transperineal MRI/US fusion-guided prostate biopsy

**DOI:** 10.1038/s41598-024-55492-w

**Published:** 2024-03-07

**Authors:** Viktor Alargkof, Christian Engesser, Hanns Christian Breit, David Jean Winkel, Helge Seifert, Pawel Trotsenko, Christian Wetterauer

**Affiliations:** 1 Department of Urology, University Hospital Basel, Spitalstrasse 21, 4056 Basel, Switzerland; 2 Department of Radiology, University Hospital Basel, Basel, Switzerland; 3 Department of Urology, Landesklinikum Wiener Neustadt, Wiener Neustadt, Austria; 4 University of Basel, Basel, Switzerland; 5 Department of Medicine, Faculty of Medicine and Dentistry, Danube Private University, Krems, Austria

**Keywords:** Prostate cancer, Urology, Prostate

## Abstract

Transperineal fusion prostate biopsy has a considerable learning curve (LC). Robotic-assisted transperineal MRI/Ultrasound fusion-guided biopsy (RA-TP-FBx) may have an easier LC due to automatization. We aimed to assess the LC of RA-TP-FBx and analyze its most difficult steps. We prospectively analyzed cases randomized to a biopsy-naïve urology resident, the chief resident, and an expert urologist in RA-TP-FBx (controls). We also analyzed consecutive cases in the LC of the expert. The LC was defined by procedure time, PCa detection rate (including stratification by PI-RADS), entrustable professional activities (EPA) assessment scores, and the NASA task load index. We collectively performed 246 RA-TP-FBx with the Mona Lisa device. Procedure time for residents decreased steeply from maximum 53 min to minimum 10 min, while the mean procedure time for the expert was 9 min (range 17–5 min). PCa detection for PI-RADS-4 lesions was 57% for the naïve resident, 61% for the chief resident and 62% for the expert. There was also no difference in Pca detection for PI-RADS-4 lesions when comparing the first and second half of the experts’ biopsies (*p* = 0.8). Maximum EPA score was registered after 22 cases. Workload steeply declined. Proficient RA-TP-FBx performance appears feasible after 22 cases regardless of previous experience.

## Introduction

The approach and technique of prostate biopsy have witnessed a drastic evolution in recent years. The European Association of Urology guidelines now strongly recommend transperineal over transrectal access mainly due to data suggesting significantly reduced infectious complications using the transperineal route^1^. The traditional approach of 8–12-core systematic biopsy has also been reported to miss clinically significant prostate cancer (csPCa defined here as International Society of Urological Pathology grade group: ISUP ≥ 2) in up to 30% of cases^2^. With multiparametric magnetic resonance imaging (MRI) having become a mainstay in prostate cancer (PCa) diagnostics and MRI-targeted approaches being reported to increase the detection of csPCa^3^, MRI/Ultrasound (US) fusion biopsies of the prostate now constitute the state-of-the-art^4^.

As the latest development, novel robotic-assisted transperineal platforms promise high precision planning and execution of new sampling strategies such as target saturation^5,6^ and perilesional biopsies^7^, offering the potential to further increase diagnostic accuracy, while maintaining low complication rates by performing all biopsies through only two puncture sites at the perineum^8,9^. Highly precise PCa mapping, with the ability to store and rapidly revisit biopsied sites using robotic platforms, is also investigated as a tool for focal treatments^10^.

The learning curve (LC) is an impactful factor when it comes to prostate biopsy. In the ever-recurring debate of transrectal versus transperineal biopsy, proponents of transrectal often argue for an easier LC^11^. In some practices, the LC may even be a parameter halting the full transition from transrectal to transperineal^12^. Knowledge of the LC is also important in the research setting and studies investigating outcomes of prostate biopsy are increasingly utilizing robotic-assisted platforms^13–15^. Particularly when investigating sampling strategies, information on operator experience should be stated to provide a reference when reporting research outcomes^16^.

While a significant body of research exists about the LC for transrectal and transperineal MRI/US fusion-guided prostate biopsy^16–20^, to the best of our knowledge, there are no reports regarding the LC for robotic-assisted transperineal MRI/US fusion-guided biopsy of the prostate (RA-TP-FBx). Robotic assistance in needle guidance with automated control of the needle pathway and penetration depth could provide an advantage over potentially less precise human targeting, not only for reducing missing identified regions of interest but also for precisely executing more elaborate sampling strategies that require exact biopsy placement in very adjacent, anterior, and peripheral areas of the prostate. Ultimately, the definition of an LC for robotic-assisted prostate biopsy is necessary for centers where it is performed to build an efficient training program and to ensure institutional quality standards are met by providing references to avoid cancer underdetection and exposure of patients to overly prolonged operation times.

The primary objective of this study was to prospectively assess the LC of RA-TP-FBx. The secondary objective was to identify and analyze the tasks/steps of RA-TP-FBx, which are the most time-consuming and have the highest degree of difficulty.

## Subjects and methods

### Patient selection, trainees, and supervisor

We performed a prospective analysis of the RA-TP-FBx of 91 men who underwent the procedure at our institution from January to December 2022 due to elevated prostate-specific antigen (PSA) values, suspicious lesions on MRI (Prostate Imaging–Reporting and Data System v2.1, PI-RADS, score ≥ 3) or digital rectal examination, as part of active surveillance (AS) and as part of a bi-parametric MRI PCa screening trial: VISIONING^21^. All patients provided written informed consent. The study was approved by the local ethics commission (ID 2020-01381) and was carried out per the Declaration of Helsinki. The cases were randomly assigned to 3 operators: (1) a first-year urology resident (1R), (2) the urology chief resident (CR), and (3) an expert (EX) urologist in RA-TP-FBx. At the beginning of the study, 1R had no previous prostate biopsy experience. The CR had no robotic biopsy experience and no transperineal biopsy experience but had completed 30 cases of transrectal prostate biopsy. The EX was the institution's most experienced surgeon in prostate biopsy, having completed 300 cases of RA-TP-FBx and 200 free-hand transperineal cognitive fusion biopsies. The residents did not receive any training for the robotic device before the first case. All procedures performed by residents were assisted or supervised and evaluated by the expert. Randomization was performed using the block method to facilitate real-life conditions and reduce the risk of bias in the allocation of cases per operator.

To assess for changes in cancer detection over a longer time period, we further performed an analysis of the first 155 consecutive RA-TP-FBx performed by the expert. The biopsy indications and ethics considerations described for the multi-surgeon cohort were also valid for the single-surgeon analysis.

### 3‑D modeling, equipment, biopsy technique, and histological analysis

Suspicious lesions on MRI were classified according to PI-RADS v2.1 and marked on a 3-D model of the prostate, created by dedicated uroradiologists. The biopsy was performed with an iSR'obot™ MonaLisa device (Biobot©) under general anesthesia. Antibiotic prophylaxis was omitted in the absence of a specific indication, based on data demonstrating zero de novo infectious complications using only skin disinfection^8^. Routine placement of a transurethral catheter, bowel preparation, or enemas were omitted.

The Mona Lisa device uses a software-controlled robotic arm attached to the operating table, which autonomously determines needle placement-angle and penetration depth. The system enables puncture of the perineum through the same entry point allowing complete sampling via only two puncture points (one per lobe). Three different tissue sampling methods were utilized in this study: (1) trizonal schema: target saturation biopsies + HALO biopsies (i.e. perilesional biopsies) + systematic biopsies for cases with lesions with PI-RADS score of ≥ 3, (2) systematic-only biopsies: in patients without suspicious PI-RADS lesions but with another strong biopsy indication (suspicious digital rectal exam or progressively elevated PSA), (3) targeted-only biopsies: as part of the VISIONING trial^21^. Histological evaluation was performed by specialized uropathologists.

### Definition of learning curve

The learning progress was evaluated via four pillars: (1) efficiency—measured by overall time as well as time for completion of individual steps of the procedure (equipment and patient positioning check, software loading, transrectal probe positioning, prostate scanning and 3-D-modelling, fusion and biopsy planning, tissue-taking with biopsy-gun), (2) effectiveness—defined as the detection rate of prostate cancer (PCa) including stratification by PI-RADS score, (3) operator and supervisor performance evaluation—measured via validated entrustable professional activities (EPA) questionnaires^22–24^, which were filled out in an independent and blinded fashion by the trainees and the expert as well as an overall learning behavior description which was decided in consensus between expert and trainee (as described in Situational Leadership theory by Hersey et al.)^23^, (4) workload—measured by the NASA task load index^25^

### Data collection and analysis

Demographic, clinical, and histopathological data were gathered and analyzed. Statistical analyses were performed with SPSS Statistics 24.0 (IBM©). The database was set up using Excel (Microsoft©). Fisher’s exact test was used to compare nominal data. The Student’s t-test (dependent/independent) was applied to determine significant differences among the normally distributed data. Logistic regression was used for binary classification, i.e., to estimate the posterior probability of a binary response based on a list of independent predictor variables. This probability is described by a generalized linear model. Odd`s ratio (OR) was performed for risk assessment. All calculations were performed at a two-sided significance level of α = 0.05.

## Results

There was no significant difference in patient characteristics between operators, which might have influenced the outcomes. Patient characteristics are summarized in Table 1. Patient characteristics for the single-surgeon cohort are summarized in the supplementary Table S1.1.

**Table 1:** Baseline characteristics.

Parameter	All patients n = 91	1st y—resident n = 35	Chief resident n = 21	Expert n = 35
	*Median (IQR)*			
Age (years)	64 (59–70)	62 (59–69)	67 (56–72)	65 (59–69)
PSA (ng/ml)	5.6 (3.4–8.3)	5.7 (3.7–8.2)	4.6 (2.7–7.2)	5.6 (3.9–8.9)
Prostate volume (cm^3^)	47 (32–65)	45 (32–57)	46 (32–60)	50 (33–71)
Number of biopsies	23 (19–29)	25 (19–28)	21 (19–31)	21 (19–27)
Diameter of lesions (mm)	9 (6–13)	11 (6–13)	8 (7–16)	9 (6–13)
	*n (%)*			
Previous biopsy	20 (22)	10 (28.6)	3 (14.3)	7 (20)
Under AS	8 (8.8)	4 (11.4)	2 (9.5)	2 (5.7)
Suspicious DRE	12 (13.2)	5 (14.3)	4 (19)	3 (8.6)
Positive family history	7 (7.7)	2 (5.7)	4 (19)	1 (2.9)
PI-RADS 3	9 (9.9)	4 (11.4)	2 (9.5)	3 (8.6)
PI-RADS 4	55 (60.4)	21 (60)	13 (61.9)	21 (60)
PI-RADS 5	18 (19.8)	5 (14.3)	5 (23.8)	8 (22.9)

Parameter	RS versus EX	CR versus 1R	CR versus EX	1R versus EX
	*p* Value			
Age (years)	0.44	0.32	0.34	0.47
PSA (ng/ml)	0.29	0.15	0.44	0.12
Prostate volume (cm^3^)	0.44	0.49	0.45	0.44
Number of biopsies	0.13	0.41	0.21	0.13
Diameter of lesions (mm)	0.12	0.45	0.20	0.13

*1R* 1st year resident, *CR* chief resident, *EX* expert, *RS* residents, *PSA* prostate-specific antigen, *AS* active surveillance, *DRE* digital rectal examination, *PI-RADS* prostate imaging-reporting and data system v2.1.

**Figure 1:**
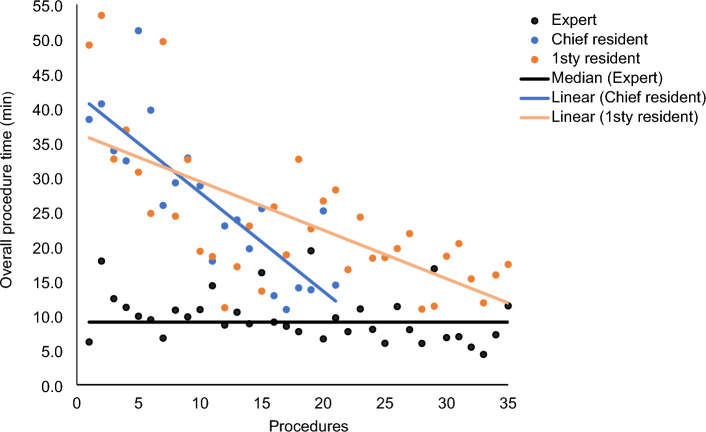
Evolution of overall procedure time per operator.

The mean procedure time (range) for 1R was 20 min (11–53) versus 25 min (10–40) for CR versus 9 min (5–17) for EX. The main difference between residents and the expert stemmed from the ultrasound probe positioning (mean time for EX: 0.4 min vs. 1R: 3.3 min vs. CR: 4 min) and biopsy-gun time (mean time for EX: 4.2 min vs. 1R: 9.9 min vs. CR: 10.3 min). The evolution of overall procedure time per operator is shown in Fig. 1.

Detailed prostate biopsy results are shown in Table 2. Overall, PCa was detected in 61% and csPCa in 35% of biopsies. PCa and csPCa detection rates per operator on patient level stratified by PI-RADS are shown in Table 3. There was no statistically significant difference regarding cancer detection between operators (1R vs. CR OR 2.1; 95% confidence interval [CI] 0.6–6.6; *p* = 0.2; 1R vs. EX OR 0.7; 95% CI 0.2–1.8; *p* = 0.4, CR vs. EX OR 1.4, 95% CI 0.4–4.7; *p* = 0.5). In the single-surgeon cohort, a total of 154 biopsies were performed. In this cohort, PI-RADS 4 lesions were the most numerous, being present in 104 cases (52.5%) (53 cases in the first half and 51 in the second half), followed by 59 cases with PI-RADS 3 lesions (29.8%) and 35 cases with PI-RADS 5 lesions (17.7%). There was no change in cancer detection for PI-RADS-4 lesions when comparing the first 77 to the latter 78 experts’ biopsies (OR 0.9; 95% CI 0.4–2; *p* = 0.8).

**Table 2:** Biopsy results.

	1st y—resident n = 35	Chief resident n = 21	Expert n = 35
Targeted-only cases (%)	5 (14.3)	1 (4.8)	3 (8.6)
Trizonal-schema cases (%)	25 (71.4)	19 (90.5)	29 (82.9)
Systematic-only cases (%)	5 (14.3)	1 (4.8)	3 (8.6)
PCa in targeted-only cases (%)	4 (80)	1 (100)	2 (66.7)
csPCa in targeted-only cases (%)	4 (80)	1 (100)	2 (66.7)
Pca in trizonal-schema cases (%)	13 (52)	13 (68.4)	19 (65.5)
csPca in trizonal-schema cases (%)	8 (32)	11 (57.9)	14 (48.3)
Pca in systematic-only cases (%)	2 (40)	1 (100)	1 (100)
Overall Pca detection (%)	19 (54.3)	15 (71.4)	22 (62.9)
Overall csPCa detection (%)	13 (37.1)	13 (61.9)	16 (45.7)
Total number of cores	847	496	765
Mean cores per Bx (range)	24 (5–42)	23 (6–40)	21 (7–37)
Pca cores (%)	123 (14.5)	135 (27)	122 (15)
Systematic cores with non-prostate tissue (n per biopsy)	18 (0.5)	13 (0.6)	14 (0.4)
ISUP I	6	2	6
ISUP II	9	8	14
ISUP III	1	4	2
ISUP IV	2	0	0
ISUP V	1	1	0
RP after biopsy	5	6	11
Matching ISUP in RP (Biopsy vs. RP Specimen) (%)	4 (80)	4 (66.7)	8 (72.7)

*PCa* prostate cancer, *csPCa* clinically significant PCa, *BX* biopsy, *ISUP* international society of urologic pathology, *RP* radical prostatectomy.

**Table 3:** Prostate cancer detection rates per operator on patient level stratified by PI-RADS.

Operator	PI-RADS	Patients	Patients with PCa	Patients with csPCa
		*n (%)*		
All (n = 91)				
	3	9 (9.9)	6 (66.7)	4 (44.4)
	4	55 (60.4)	33 (60)	23 (41.8)
	5	18 (19.8)	13 (72.2)	13 (72.2)
1R (n = 35)				
	3	4 (11.4)	2 (50)	1 (25)
	4	21 (60)	12 (57.1)	8 (38.1)
	5	5 (14.3)	3 (60)	3 (60)
CR (n = 21)				
	3	2 (9.5)	1 (50)	1 (50)
	4	13 (61.9)	8 (61.5)	6 (46.2)
	5	5 (23.8)	5 (100)	5 (100)
EX (n = 35)				
	3	3 (8.6)	3 (100)	2 (66.7)
	4	21 (60)	13 (61.9)	9 (42.9)
	5	8 (22.9)	5 (62.5)	5 (62.5)
RS (n = 56)				
	3	6 (10.7)	3 (50)	2 (33.3)
	4	34 (60.7)	20 (58.8)	14 (41.2)
	5	10 (17.9)	8 (80)	8 (80)

*PI-RADS* prostate imaging-reporting and data system v2.1, *PCa* prostate cancer, *csPCa* clinically significant prostate cancer, *1R* 1st year resident, *CR* chief resident, *EX* expert, *RS* residents.

Assessment of EPA Questionnaires revealed that 1R was able to individually carry out all steps of the procedure without guidance or interference from EX, consistently as of the 15th and CR as of the 14th procedure. Before this threshold, it was required that EX interfere with course correcting or actively taking over single steps of the procedure, and the biopsies were marked as individually incomplete. Maximum self-rating was registered as of the 22nd procedure for 1R and the 16th procedure for the CR. Maximal EX rating and joint evaluation as proficient were consistently awarded as of the 22nd biopsy for 1R and as of the 17th for CR.

Residents’ assessment of the difficulty rating of tasks was unequivocal. Ultrasound probe positioning was identified as the most difficult task followed by scanning/3-D modeling of the prostate. Fusion and biopsy planning was the third most difficult task and tissue-taking with the biopsy-gun was ranked last.

**Figure 2:**
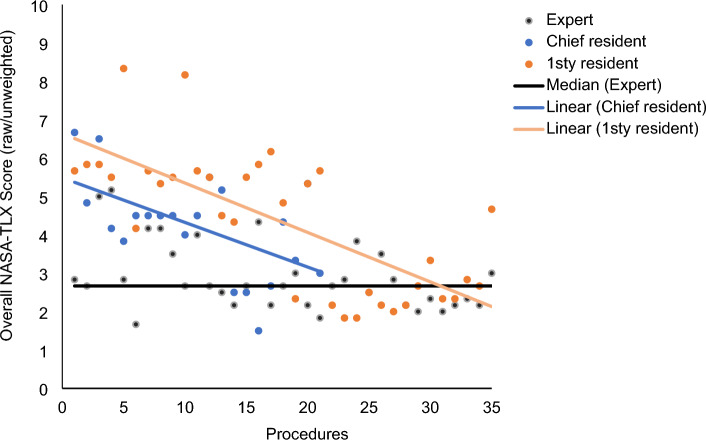
Evolution of overall NASA-TLX Score (raw/unweighted) per operator.

A raw/unweighted overall score per operator was calculated via the NASA Task load index questionnaires. The mean overall score was 4.3 points for 1R versus 4.2 for CR versus 2.9 for EX. The residents displayed a higher score than the expert (*p* < 0.001). Workload decreased steeply with increasing experience. The evolution of the overall NASA score per operator is shown in Fig. 2.

## Discussion

MRI and targeted-biopsy-only may increase csPCa detection while decreasing ISUP 1 overdetection and omitting systematic biopsies is a matter of consideration in the screening setting^26^. However, studies indicate a considerable LC for cancer detection when utilizing target fusion^16–20^. Strategies to reduce error margin in the diagnostic chain include the double reading of MRIs by expert uroradiologists^26^, integration of artificial intelligence in MRI interpretation^27^, and novel tissue sampling strategies such as target saturation and perilesional biopsies^5–7^. In any case, a central factor to be considered when thinking of discarding the “safety net” of systematic biopsies—which may have been “catching” correctly identified cancers on MRI that were missed on targeted biopsy (TBx)-, is the accuracy of the operator performing the biopsy. It has been hypothesized that the user-friendliness and automatization of robotic assistance could decrease the LC and interoperator variability^12^. We thus aimed to investigate the LC of RA-TP-FBx to also understand its place in this forever changing prostate-biopsy-world, future urology residency programs, and potentially increased demand for prostate biopsies to come, as the prostate cancer screening landscape changes^28^.

Published studies on the LC of MRI-TBx have used different methodologies so far. The presence of an LC was alluded to, in the study of Calio et al.^16^, who segregated 1528 biopsies in tertials and identified a significant increase in PCa detection rate in TBx with increasing operator experience. Similarly, in the study of Gaziev et al.^17^, a PCa detection increase of 36% was reported, when comparing the first and second half of 140 transperineal fusion biopsies. Subsequent single-surgeon studies attempted to quantify the LC. In the study of Kasabwala et al.^18^, an LC of 98 cases was identified by studying the distance difference between planned and actual core trajectories in a cohort of 173 biopsies performed with the Artemis platform. Halstuch et al.^19^ reported an LC of 125 cases for transperineal fusion biopsy using a combination of procedure time and PCa detection rate for PI-RADS 3 lesions in a series of 256 biopsies. Finally, in the novice versus expert study of Mager et al.^20^, it was suggested that at least 63 fusion biopsies are needed for a novice trainee to reach expert level in terms of TBx cancer detection and procedure time.

The study design and parameters used to define the LC of any prostate biopsy technique pose a significant challenge, which is mirrored in the heterogeneity of methodologies used in LC studies to date. Prostate biopsy is in essence a diagnostic test and as such the main outcome measure is the cancer detection rate. However, the detection rate is influenced by multiple factors such as MRI performance technique and interpretation^29^, 3-D modeling and fusion^30^, intraprostatic and intralesional tumor heterogeneity, biopsy technique, and finally histopathological interpretation^31^. Ultimately, the only tool to accurately assess cancer detection would be to obtain radical prostatectomy specimens after all biopsies, positive and negative alike: a truly radical and unethical notion. Single-surgeon results are dependent on prior experience, and potentially surgical skill and may not mirror those of other operators. And lastly, procedure time may be affected by the prostate size, anesthesia, or patient tolerance factors, and may not always coincide with how diagnostically successful an operation was.

We aimed to compensate for these limitations via the design of the study. We considered that principally, trainees are deemed proficient in a procedure when they present subjective confidence to individually complete all its steps and are also assessed as capable by a supervising mentor^32^. Efficiency, effectiveness, the required workload, and safety are the rest of the fundamental markers of proficiency. We consider biopsy complications such as hematospermia, hematuria, and urinary retention as minorly related to surgical expertise, as they are primarily affected by patient factors (e.g. baseline urinary symptom scores) and based on the previously reported excellent safety profile of robotic biopsy^8,9^, elected not to include complications in this work. We thus decided to analyze operation time, cancer detection rate, and the outcomes of validated EPA and NASA-TLX questionnaires in a real-life prospective design.

We opted for an analysis with different levels of operator experience as well as controls to test if transferable skills from experience with other biopsy techniques could have an impact on the LC and to evaluate if it is possible to describe a single LC that can apply to the most operators possible regardless of previous experience. Comparing two trainees also allowed to assess potential differences in individual surgical skill and individual learning speed. For example, the CR interestingly displayed longer operation times at the beginning of the learning curve than the 1R. The validated surgical assessment tool of entrusted professional activities (EPA), which has been internationally adopted by surgical training programs to assess the learning curve for surgical techniques^22,24^, was selected to rate observed performance. General anesthesia was performed in all cases, harmonizing the procedure by eliminating patient factors such as individual pain tolerance and movement which may have introduced further bias in affecting biopsy effectiveness and time. Simultaneously, a pragmatic approach was followed since biparametric, multiparametric, 1.5 T, and 3 T MRIs with or without coil and different sampling strategies were allowed. The inclusion of different sampling techniques simulated real-world conditions, posing a different challenge to operators, disrupting operator automatisms carried on from one case to the next, and reducing “forgetting” in the learning curve by avoiding long waiting times between cases. Finally, quality control was implemented for the aspects of imaging and pathology during the whole study through exclusive cooperation with a central, constant team of dedicated uroradiologists and uropathologists for respective MRI interpretation, prostate segmentation, and histopathological analysis.

Certain limitations remained. The findings for the Mona Lisa platform may not apply when considering the technical particularities of other platforms. Furthermore, performance heterogeneity of even dedicated uroradiologists and uropathologists has been reported^29,31^ and may have influenced the results of cancer detection. A stratification for patient characteristics and sampling strategy in the randomization process was omitted. However, this was a deliberate design as multiple parameters can have an impact on different aspects of the learning curve, e.g. tissue sampling methods can affect biopsy-gun time, PSA and age can affect PCa detection rates, prostate size can affect the total number of biopsies and in this way modeling time and overall procedure time, etc. Adjusting for every potential confounder would not allow for a pragmatic approach to answering the main scientific question of what the learning curve of this novel robotic technique is in daily practice. Lastly, generalizability may be influenced by the mentoring quality in each training program.

Our study suggests that accurate, independent, and confident performance of RA-TP-FBx with near-expert-level operation time and low workload demand is possible for residents regardless of previous experience as of 22 procedures. Regarding effectiveness, we did not observe any difference in PCa detection between operators. Most notably we identified an almost identical csPCa detection rate for cases with PI-RADS 4 lesions, which were evenly distributed between operators. We consider the detection rate in PI-RADS 4 lesions to be the most appropriate to evaluate for biopsy accuracy given the high probability of true negatives in PI-RADS 3 lesions and the low probability of low accuracy in PI-RADS 5 lesions. Finally, considering the frequently used methodology of other authors comparing cancer detection rate in chronologic blocks, we also analyzed the detection rate for PI-RADS 4 lesions (which were the most numerous, i.e. providing the most adequate statistical power and displayed even distribution between the time blocks) in the first and second half of the experts' biopsies and found no difference in cancer detection over time.

Regarding individual procedure steps, positioning the transrectal probe was particularly challenging for both the 1R and the CR. This difficulty arose from the urge to visualize the whole prostate upon initial positioning. Instead, placement of the probe as dorsally and as deeply as possible with a subsequent ventral tilt of the tip was noted to provide the most time-efficient placement and ensure full range coverage of the gland. While probe positioning was learned as quickly for both residents, scanning and modeling time decreased more steeply for the CR, potentially owing to more extensive experience with transrectal ultrasonography of the prostate. Biopsy-gun-time was also longer for both 1R and CR and could be further reduced by approving the next biopsy position while unloading the gun and inspecting the specimen of the core previously taken. These tasks can easily be carried out while the robot is positioning for the next biopsy site. Despite the reduction of procedure time for the residents, more cases may be required to achieve consistent expert-level procedure time, which however was shorter in this study than previously reported for robotic biopsy^33^.

In terms of contextualizing these findings in the prostate biopsy landscape, we noted a considerably easier learning curve for the robotic platform compared to what has been described for other transperineal prostate biopsy techniques (range of 63–125 cases)^16–20^. In summary, we observed that once correct probe positioning and marking of the prostate boundaries were learned, the remaining procedure steps appeared very easy due to the simplification offered by robotic assistance. Particularly, reaching the regions of interest, which may be a pitfall for targeting failure in other biopsy techniques, appeared easier for the robotic platform due to the automatized needle pathway and needle penetration depth selection.

Based on our results, we consider that as of 22 cases proficiency in RA-TP-FBx was reached, meaning the ability to consistently, successfully troubleshoot and fully autonomously, and confidently perform the procedure with low effort, within a standard operative time, and with uncompromised biopsy quality. We presume steady biopsy accuracy due to the automatization offered by robotic technology. Nevertheless, further studies are required to validate these findings.

### Supplementary Information

Supplementary Information.

## Data Availability

The datasets supporting the results of the present study are available upon reasonable request from the corresponding author.
