# Supplementary material for: The learning curve for robotic-assisted transperineal MRI/US fusion-guided prostate biopsy
Source: Sci Rep. 2024 Mar 7;14:5638. doi: 10.1038/s41598-024-55492-w (PMC10920700; doi:10.1038/s41598-024-55492-w)
Supplement: Supplementary file 1 — Supplementary Information. [file 41598_2024_55492_MOESM1_ESM.docx]

| Parameter | All patients  n = 155 |
| --- | --- |
|  | Median (IQR) |
| Age (years) | 64.4 (57.7 – 70.5) |
| PSA (ng/ml) | 5.6 (4.2 – 7.8) |
| Prostate volume (cm^3^) | 40 (30.3 – 54) |
| Number of biopsies | 28 (20 – 31) |

**Table 1.1** Baseline characteristics in the single-surgeon cohort
